# Efficacy of an Antibiotic Loaded Ceramic-Based Bone Graft Substitute for the Treatment of Infected Non-Unions

**DOI:** 10.3390/biomedicines10102513

**Published:** 2022-10-08

**Authors:** Holger Freischmidt, Jonas Armbruster, Catharina Rothhaas, Nadine Titze, Thorsten Guehring, Dennis Nurjadi, Jan Philippe Kretzer, Gerhard Schmidmaier, Paul Alfred Grützner, Lars Helbig

**Affiliations:** 1 Department of Trauma and Orthopedic Surgery, BG Klinikum Ludwigshafen at Heidelberg University Hospital, 67071 Ludwigshafen am Rhein, Germany; 2 Trauma Centre, Hospital Paulinenhilfe Stuttgart at Tübingen University Hospital, Rosenbergstr. 38, 70176 Stuttgart, Germany; 3 Department of Infectious Diseases, Medical Microbiology and Hygiene, Heidelberg University Hospital, Im Neuenheimer Feld 324, 69120 Heidelberg, Germany; 4 Department of Infectious Diseases and Microbiology, University of Lübeck, Ratzeburger Allee 160, 23538 Lübeck, Germany; 5 Laboratory of Biomechanics and Implant Research, Clinic for Orthopedics and Trauma Surgery, Heidelberg University Hospital, Schlierbacher Landstrasse 200a, 69118 Heidelberg, Germany; 6 Clinic for Trauma and Reconstructive Surgery, Center for Orthopedics, Trauma Surgery and Spinal Cord Injury, Heidelberg University Hospital, Schlierbacher Landstraße 200a, 69118 Heidelberg, Germany

**Keywords:** ceramic-based bone graft substitute, animal model, non-union, bone defect, bone infection, osteitis

## Abstract

The treatment of non-unions is often complicated by segmental bone defects and bacterial colonization. Because of the limited availability of autologous bone grafts, tissue engineering focuses on antibiotic-loaded bone graft substitutes. HACaS+G is a resorbable calcium sulphate-hydroxyapatite loaded with gentamicin. The osteoinductive, osteoconductive, and anti-infective effect of HACaS+G has already been demonstrated in clinical studies on patients with chronic osteomyelitis. However, especially for the treatment of infected non-unions with segmental bone defects by HACaS+G, reliable clinical testing is difficult and sufficient experimental data are lacking. We used an already established sequential animal model in infected and non-infected rat femora to investigate the osteoinductive, osteoconductive, and anti-infective efficacy of HACaS+G for the treatment of infected non-unions. In biomechanical testing, bone consolidation could not be observed under infected and non-infected conditions. Only a prophylactic effect against infections, but no eradication, could be verified in the microbiological analysis. Using µ-CT scans and histology, osteoinduction was detected in both the infected and non-infected bone, whereas osteoconduction occurred only in the non-infected setting. Our data showed that HACaS+G is osteoinductive, but does not have added benefits in infected non-unions in terms of osteoconduction and mechanical bone stability, especially in those with segmental bone defects.

## 1. Introduction

Non-unions have been a great challenge for orthopaedic surgeons and their patients for a long time. They have a large socio-economic impact due to high costs, as well as pain and a long-lasting healing process for the patients [1]. Non-union rates vary between 1.7% in the femoral shafts after reamed intramedullary nailing [2] of up to 18.5% in tibial fractures [3]. In the case of complex or open fractures, the risk of infection is up to 55% [4,5,6]. These injuries often result in delayed bone healing and large bone defects [7,8].

Fracture-related infections (FRI) and osteomyelitis are most commonly caused by *Staphylococcus aureus* [7,9,10,11,12].

Standard treatment for non-unions consists of repeated surgical debridement, sufficient fixation, bone reconstruction, and antibiotic therapy [13]. Autologous bone grafting is still the gold standard for healing bone defects. Although there are some limitations regarding autologous bone graft, as an additional surgical intervention, with its possible pain and complications, as well as the limit in quantity, it represents the current gold standard of non-union treatment [1,14]. Therefore, there is an increasing need for bone graft substitutes to treat large segmental bone defects, especially when there is an infectious component. There are many approaches, such as allograft bone matrix, stem cells, and synthetic materials combined with different adjuvants and carriers [1,15].

Cerament G (Bonesupport Holding, Lund, Sweden) consists of hydroxyapatite (HA), calcium sulphate (CaS), and gentamicin. HA is slowly resorbed and gives a good stability, while CaS is resorbed more quickly positively impacts bone remodelling through its osteoconductivity [16,17]. In addition, Cerament G has an antibiotic component through gentamicin, which serves as prevention and therapy for bone infections [18,19]. As it is only locally applied throughout the HA-CaS carrier, the plasma level is far lower than for any systemic antibiotics. This means a better tolerance, especially regarding nephro- and ototoxicity. Still, the local antibiotic levels are far above the minimum inhibitory concentration [20].

Therefore, Cerament G combines two important aspects of bone healing—osteoconductive strength and antibiotic effects—which makes it a promising alternative for treating large (infectious) bone defects [15,16]. A crucial advantage for using CERAMENT is that it forms a paste that can be injected into bone defects, resulting in complete filling of the cavity and closing out any dead space, obliterating any areas that might contain residual bacteria or small segments of biofilm [19,21]. In addition, some studies have attributed an osteoinductive potential to Cerament G [21,22]. This has only been verified in µ-CT analyses, and evidence at a cellular level is still lacking. Animal studies, as well as clinical studies of single groups have already shown some promising results regarding bridging, prosthesis fixation, coating implants, and osteomyelitis treatment [19,23,24,25,26,27,28]. Because of heterogeneity in patient populations with different bone defects, comorbidities and infections, it is hard to conduct randomized comparative trials. Furthermore, detailed analysis in clinical settings is limited, which results in a lack of comparable, reliable, and valid data on its efficacy.

Our research group previously established a new sequential animal model for infection-related non-unions with segmental bone defects [29]. This two-staged animal model reflects an authentic clinical situation and allows for evaluating therapeutic interventions with bone substitutes [30] for the treatment of infected non-unions, and is thereby the first evaluation of Cerament G in an animal model of infected and non-infected non-unions. The aim of this study was to evaluate Cerament G as a therapeutic option in the treatment of infected non-unions regarding its osteoinductive, osteoconductive, and anti-infective properties by means of a standardized non-union model.

## 2. Materials and Methods

### 2.1. Preparation of Infecting Agent

*Staphylococcus aureus* subsp. *aureus* ATCC^®^ 49,230, which is already established in various animal models, was used for in-vivo infection [30,31,32,33]. As previously described, the pathogen was cultured in tryptic soy broth (TSB) at 37 °C with 5% CO_2_ under constant shaking (200 rpm). After harvesting at the mid-log phase on the day of infection, the bacterial pellet was washed with sterile phosphate buffered saline (PBS) and resuspended in sterile PBS to achieve 0.5 MFU (McFarland Unit). This contained 1.5 × 10^8^ CFU/mL and was subsequently diluted to 1 × 10^5^ CFU/mL. Then, 10 μL of this bacterial suspension including 10^3^ CFU was used for in vivo infection, as described below [30].

### 2.2. Calcium Sulfate/Hydroxyapatite Bone Graft Substitute with Gentamicin

The synthetic bone graft substitute Cerament G (Bonesupport Holding, Lund, Sweden) was prepared directly before application to the defect osteotomy, according to the manufacturer’s instructions. It consists of a powder component (40% hydroxyapatite and 60% calcium sulfate) and a liquid component (saline and gentamicin), which must be combined to achieve a final concentration of 17.5 mg gentamicin/mL paste.

### 2.3. Implants

Stainless steel Kirschner wires (Synthes GmbH, Umkirch, Germany) with a diameter of 1.2–1.6 mm were used for the first osteosynthesis, depending on the diameter of the femur. The second osteosynthesis was performed with an angular stable polyacetyl plate fixated to the femur with six cortical screws (RISystem AG, Davos, Switzerland) [29].

### 2.4. Groups

We included sixty-five animals in four groups (Table 1).

### 2.5. Animals, Operative Procedure, and Osteotomy Model

All of the experiments were approved by the Animal Experimentation Ethics Committee of Karlsruhe (35-9185.81/G-155/17). Sixty-five female, 3-month-old Sprague-Dawley rats (Charles River, Sulzfeld, Germany) with an average weight of 283 g were randomly divided into the four groups. The animals were kept in groups of three in type IV macrolon cages with a 12 h light/12 h dark cycle at approximately 22 °C and a humidity of 50–60%. Food and water were available ad available. The animals had an acclimatization period of 2 weeks before the first surgery was performed.

To test the effects of Cerament G on non-unions and infected non-unions, a previously established two-stage animal non-union model was used [29]. In the first procedure, an osteotomy was created and treated with K-wire osteosynthesis. After 5 weeks, a second procedure was done to treat the developed non-union with an angle-stable plate.

Before surgery, general anesthesia was performed as described before [29]. During the first surgery, a diamond disk (Dremel, Racine, WI, USA) was used to perform a 5 mm mid-diaphyseal full-thickness osteotomy on the left femora. Afterwards, the femora were fixed intra-medullary with K-wires as in a rotationally unstable nail osteosynthesis [29]. Before inserting the K-wire, 10 µL of PBS was injected into the medullary cavity in groups 1 and 3, and 10^3^ CFU *S. aureus* in 10 μL PBS was injected in groups 2 and 4.

After 5 weeks, during the second surgery, the K-wire was removed and a radical debridement was performed. Microbiological swabs were taken from the non-union region. A 25 mm long, 4 mm wide, angle-stable poly-acetyl plate (RISystem AG, Davos, Switzerland) with eight predrilled holes for angle-stable cortical screws was positioned on the anterolateral surface of the femur. Three proximal and three distal screws were used to fix the plate and create a stable plate-osteosynthesis with an approximately 5mm defect. Afterwards, the soft tissue was dried, and Cerament G was prepared. Then, 1.85 g hydroxyapatite/calciumsulphate powder and 0.8 mL gentamicin solution were combined, mixed thoroughly, and the mixture began to thicken. After 1–2 min of waiting, the desired consistency was reached and about 0.7 mL of paste was filled into the 5 mm gap. In both surgeries, the skin, fascia, and muscles were sutured in an intracutaneous technique (Resolon^®^ 4/0 Ethicon, Norderstedt, Germany). In addition, the wound margins were adapted with skin clamps (Autoclip Clips, Heidelberg, Germany).

### 2.6. Follow-Up

All of the animals received buprenorphine (0.3 mg/mL bw; Buprenovet^®^, Bayer AG, Leverkusen, Germany) as the analgesic medication perioperatively, and for the following 4 days every 12 h. After each procedure, postoperative stress was recorded using a score sheet. These included daily monitoring of body weight, general condition, spontaneous behavior in the cage, and clinical examination (temperature, respiration, pulse, and warmth of extremities). These parameters were checked daily until the animals regained their preoperative bodyweight and a good clinical condition. Furthermore, the animals were explored for clinical signs of infection of the operated limb (swelling, reddening, impairment of wound healing, and loss of passive motion in the left hind leg). Eight weeks after the second surgery, the animals were sacrificed.

### 2.7. μ-CT Scan Evaluation

All of the animals were scanned with the Skyscan 1076 in vivo microcomputed tomography scanner (Brucker, Kontich, Belgium), as previously described. A total of four scans were performed for each operated femur. The first scan was taken after five weeks, immediately before the second surgery, to evaluate the created non-union (pre-op scan). Two further in vivo scans were performed four and eight weeks after the second surgery to assess the plate osteosynthesis and bone morphology during progression. In addition, an ex vivo scan was performed at week 8 immediately after sacrifice and complete removal of soft tissue to obtain a high-resolution image at the endpoint. The detailed settings of the scans have already been described in the literature [30], as well as the parameter settings of the image reconstruction. This was performed with the SkyScan NRecon software (v.1.6.9.8, Brucker microCT, Kontich, Belgium).

A qualitative evaluation of the datasets was performed by simultaneously viewing a coronal, sagittal, and transversal plane in the SkyScan DataViewer (v.1.5.2.4, Brucker microCT, Kontich, Belgium). As previously described, the An and Friedamnn score and Lane Sandhu score were evaluated by two independent observers each [29].

Quantitative evaluation was performed using the SkyScan CTAnalyzer (v.1.13.21, Brucker microCT, Kontich, Belgium) as previously described [22,30].

### 2.8. Microbiological Evaluation

During the second surgery and when the animals were euthanized, a wound swab (Copan eSwab^TM^, Mast Diagnostics, Reinfeld, Germany) was collected from the non-union region. Then, 10 μL of the liquid was cultured on BD Columbia agar supplemented with 5% sheep blood (Becton Dickinson, Heidelberg, Germany) at 37 °C with 5% CO_2_ for 24 h. Colonies morphologically consistent with *S. aureus* were confirmed as *S. aureus* by a slide agglutination test (Pastorex Staph Plus, Bio-rad, Germany), as described elsewhere [31]. Strain typing was performed by Sanger sequencing and a comparison of the polymorphic protein A gene (*spa* typing), as previously described [31].

### 2.9. Sacrifice

Eight weeks after the second surgery, the animals were sacrificed under general anesthesia with CO_2_ in a sedation box. The left femora were accessed under sterile conditions as in previous surgeries, and a microbiological swab was obtained from the osteotomy site under sterile conditions. After disarticulating the left femur at both joints, the entire soft tissue was removed from the bone. For animals intended for biomechanical evaluation, the right femora were also dissected. Right after the μCT scanning, the bones assigned for histological evaluation were fixated in 4.5% paraformaldehyde (Roti-Histofix, Roth, Karlsruhe, Germany), and the bones intended for biomechanical testing were stored at −20 °C.

### 2.10. Mechanical Testing

For the biomechanical evaluation of the femora, a test device that measures the torsional stiffness of bones was used as previously described [32]. Two hours before the testing, the femora (control group1: 9 samples, group 2: 7 samples; intervention group 3: 7 samples, group 4: 6 samples) were left to thaw at room temperature in saline solution and the plate osteosynthesis was removed. While using a fixture device, the proximal and distal ends of the bone were placed into two embedding molds (Technovit 4071, Heraeus Kulzer GmbH, Germany) so that the defect region remained free to be tested. Afterwards, the proximal part of the femora was fixed and the distal part was restrained in a pivoting axis. While a linear constant rotation (20°/min) was applied by the testing device, the resulting maximum torque was recorded (8661–4500–V0200, Burster, Germany). When the measurement showed a stable decrease, the maximum torque of 0.5 Nm was reached or the bone fractured, and the recording was stopped. The contralateral femora were tested in the same way.

### 2.11. Histology

The femora were fixated in 4.5% paraformaldehyde (Roti-Histofix, Roth, Karlsruhe, Germany) for four days and were decalcified with ethylenediaminetetraacetic (Entkalker Soft, Roth, Karlsruhe, Germany) for 3 weeks. After withdrawal of the plates and screws, the bones went through a graded alcohol series (2 days 70% ethanol, 2 days 96% ethanol, and 2 days 100% ethanol) for dehydration. Afterwards the bones were placed in acetone for 8 h for degreasing and were finally embedded in paraffin. The femora were cut at 5 μm thickness down to the center of the sample. For overview, staining sections were treated with haematoxylin–eosin (HE) (Carl Roth GmbH & Co. KG, Karlsurhe, Germany) and Pentachrom (Chroma-Waldeck GmbH & Co. KG, Münster, Germany). Tartrat-resistent acid phosphatase (TRAP) staining (Merck KGaA, darmstadt, Germany) was used to visualize the osteoclasts. Recently formed bone was stained with Toluidine blue (Sigma-Aldrich Chemie GmbH, Steinheim, Germany). To assess vascularization and immune response, immunohistochemistry was made using anti-CD14 (ab203294, Abcam, Cambridge, UK), anti-CD31 (ab182981, Abcam, Cambridge, UK) and anti-CD68 antibodies (ab125212, Abcam, Cambridge, UK).

The stained sections were scanned from the tissue bank of the National Center for Tumor Diseases (NCT) Heidelberg. For analysis, the software Fiji ImageJ (v.1.53c) was used. Equivalent to the CT-VOI, a 6 mm ROI was selected. Using a rolling ball algorithm, the background was subtracted and a specific color threshold for each staining was applied. The selected area was measured and, to determine the proportion of stained area to total area, the total area of the bone was measured using a different threshold.

### 2.12. Statistical Analysis

Data were collated with Excel (Microsoft, Redmond, WA, USA), and GraphPad Prism version 9.4.1 (GraphPad Software, San Diego, CA, USA) was used for statistical analysis. The normal distribution was evaluated using the D’Agosino–Pearson test. One-way analysis of variance (ANOVA) followed by Tukey’s multiple comparisons test were used to detect significant differences between the four groups. The Student’s paired t-test was used to identify the differences between the two time points of the µ-CT scans. All of the tests were performed two-sided and a *p*-value < 0.05 was considered significant. Unless otherwise indicated, data are presented as mean ± standard deviation (SD).

## 3. Results

### 3.1. Failure Parameters

Two rats died in the NI control group during anesthesia during the first intervention. Two rats of the NI Cerament and one rat of the I Cerament group died during or after the second intervention. Sixty of the sixty-five animals reached the endpoint of the study.

Six animals from the NI control group and one animal from the Cerament group had to be excluded due to a secondary bone infection. One animal of the I Cerament group had to be excluded because it did not have any signs of infection, neither in the µ-CT nor in microbiological specimens at any time point. As previously described, a secondary bone infection was determined by a positive microbiological result and an An and Friedman score ≥19 [32,33,34]. Positive microbiological results with an An and Friedman score <19 were considered colonization.

Additionally, three CT scans of the I control group 4 weeks after second surgery and one CT scan of the I control group 8 weeks after second surgery were excluded due to technical errors during the imaging process.

### 3.2. Microbiologic Results Showed Only Small Anti-Infective Effects of Cerament G

In the NI control group, an increasing colonization and secondary infection rate in the microbiological specimens was delectable between the second surgery and euthanasia. This observation could also be made in an attenuated form in the NI Cerament group. All bacteria detected in the non-infected groups had a different spa-typing than the inoculated strain.

**Figure 1:**
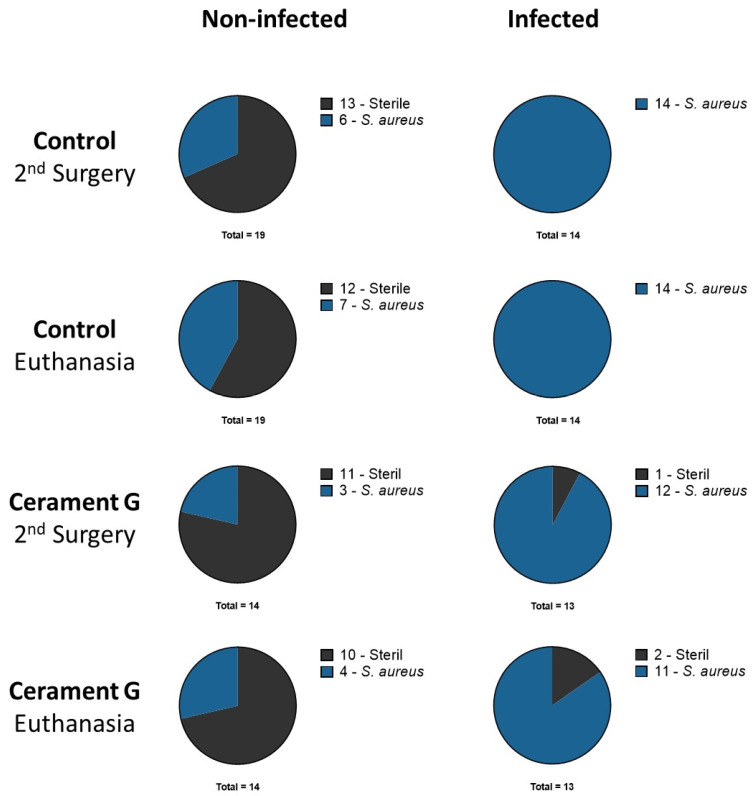
Microbiologic results of all animals after second surgery and euthanasia. Rats with secondary infection and colonization are both displayed as part of the *S. aureus* group.

All bacteria detected in the infected groups had the same *spa*-type as the inoculating strain. The inoculated *S. aureus* strain could be identified in the I-Control group in all samples at all time points. Specimens from one animal in the I Cerament group were sterile at the time of the second surgery and euthanasia. This animal was excluded from analysis because there was also no evidence of infection in the µ-CT. It must be assumed that no infection took place (Figure 1).

### 3.3. Cerament G Did Not Lead to a Stable Union in Non-Infected or Infected Conditions

**Figure 2:**
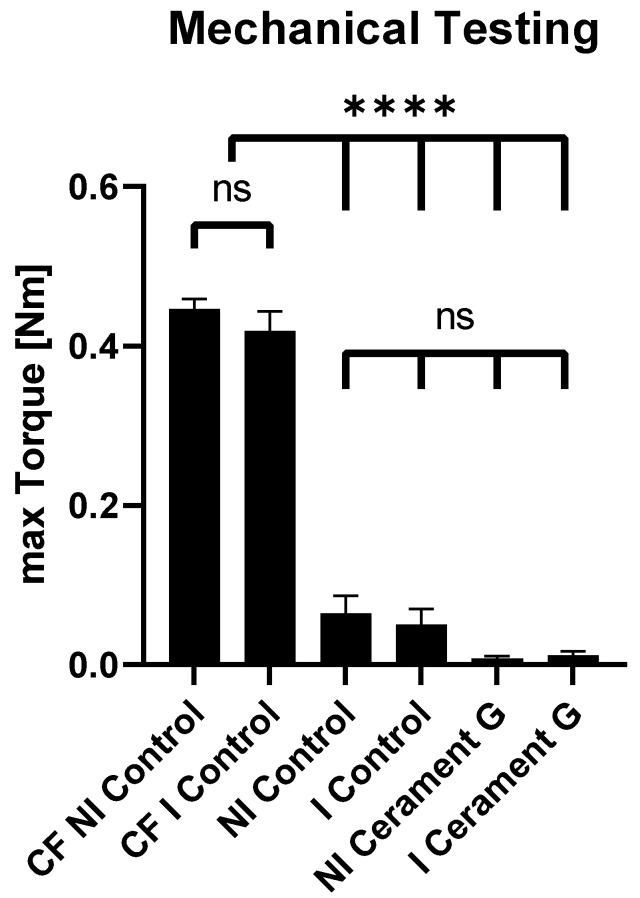
Mechanical testing. Max. torque of the non-infected and infected contralateral femora (CF NI Control + CF I Control) is significantly higher than the max. torque of all of the other groups. No significant differences between all of the other groups were detected. **** = *p* < 0.0001, ns = not significant.

For biomechanical testing, maximum torque was measured for all femora using a testing device. The contralateral femora were used as a reference for bone consolidation. The maximum torques of the infected and non-infected contralateral femora (CF NI Control, 0.45 Nm) were significantly higher than all of the other groups, whereas no significant differences could be detected in the ipsilateral femora between the control groups and the groups with Cerament G. Thus, sufficient stability in this segmental non-union model cannot be achieved by Cerament G alone (Figure 2).

### 3.4. µ-CT Analysis of the Surrounding Bone Showed an Osteoinductive Effect by Cerament G

**Figure 3:**
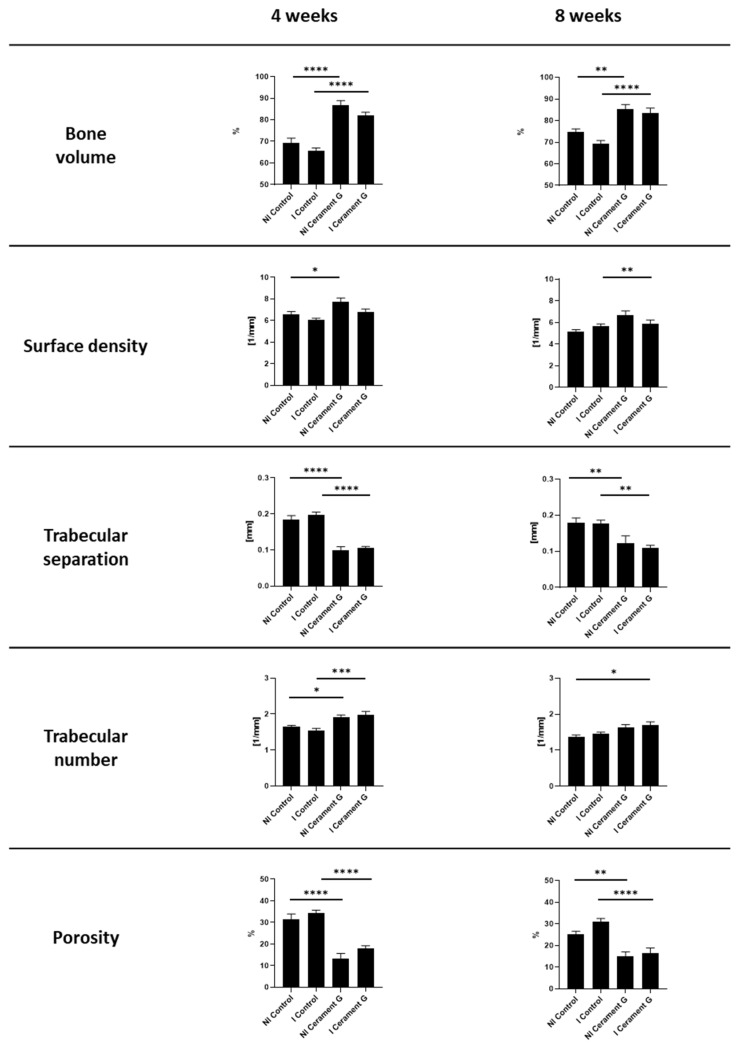
µCT Analysis of the bone 4 and 8 weeks after the second Surgery. **** = *p* < 0.0001, *** = *p* < 0.001, ** = *p* < 0.01, * = *p* < 0.05, ns = not significant.

µ-CT analysis of the surrounding bone showed a significant increase in percent bone volume four and eight weeks after the second surgery between the infected and non-infected control groups and the corresponding groups treated with Cerament G. This effect diminished for the non-infected groups after four weeks (Figure 3). A comparable situation could be observed for the bone surface density, whereas significances were found in the non-infected groups only after four weeks and in the infected groups only after eight weeks. The trabecular number is significantly increased in the groups with Cerament G four weeks after the second surgery. Osteoinduction and anti-infective prophylaxis by Cerament G was indicated by a significant reduction in trabecular separation and total porosity between the control and therapy groups four and eight weeks after the second surgery (Figure 3).

### 3.5. µ-CT Analysis of Cerament G Showed Damaging Effect of Infection

**Figure 4:**
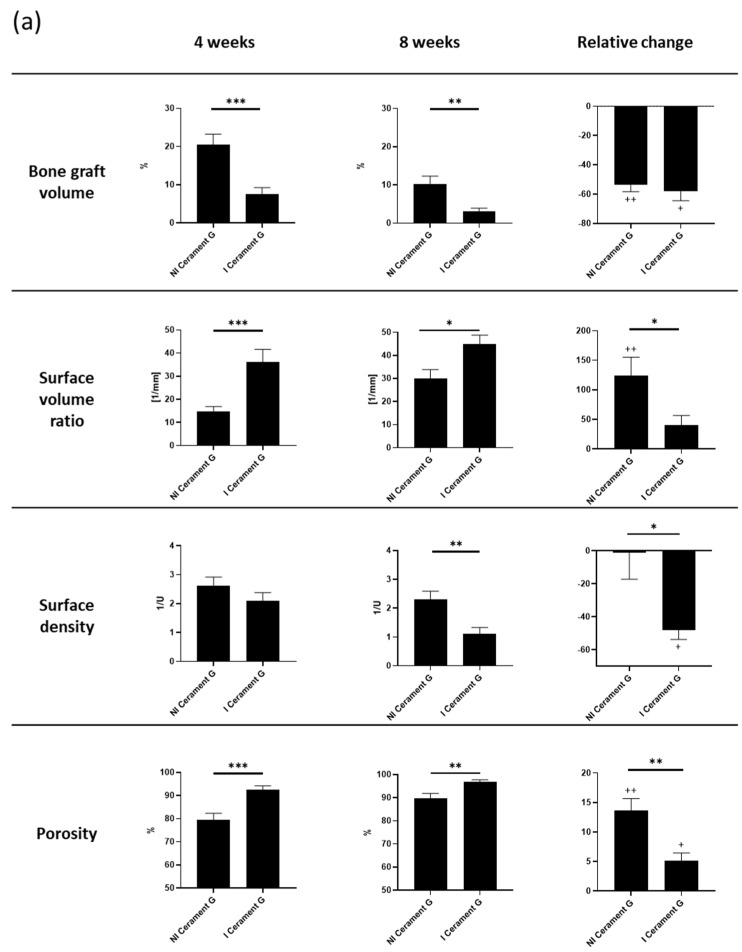

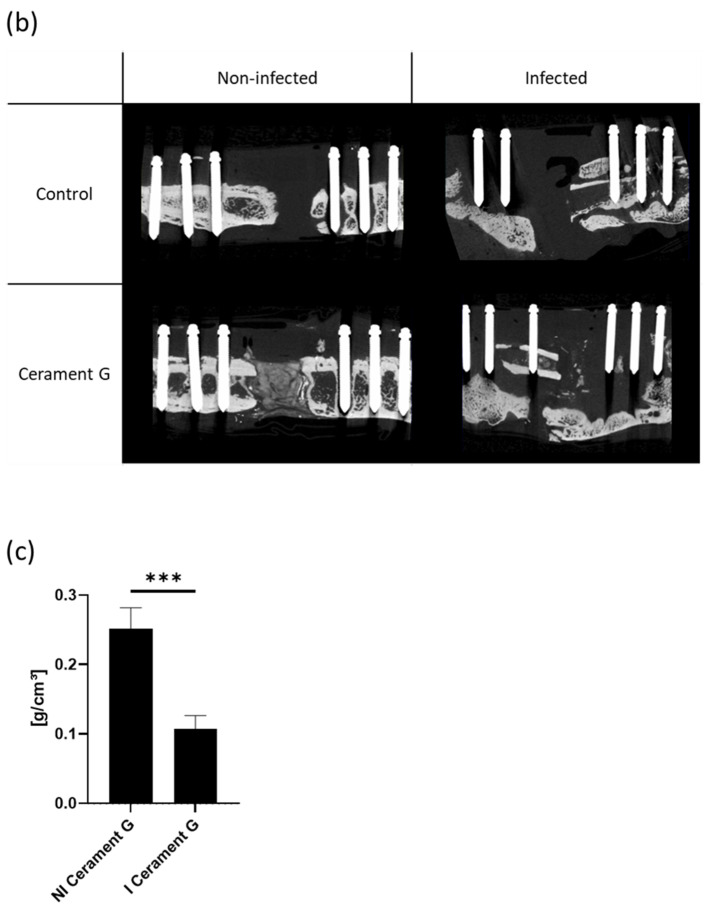
(**a**) µ-cT analysis of the bone graft four and eight weeks after the second surgery. Relative change describes the difference from the fourth to the eighth week. The relative change is measured within and between the groups. *** = *p* < 0.001, ** = *p* < 0.01, * = *p* < 0.05, ++ = *p* < 0.01 comparison of 4 to 8 weeks, + = *p* < 0.05 comparison of 4 to 8 weeks, ns = not significant. (**b**) The µ-CT images postmortem visualize the degradation of the bone graft substitute and the transformation of the surrounding bone in the infected groups. (**c**) Density of the bone graft Cerament G (BMD). The I Cerament G group shows significant less density than the NI Cerament group. *** = *p* < 0.001.

The volume of bone graft substitute decreased significantly in the infected group within eight weeks after the second surgery. The relative changes suggest that this process primarily happens in the first 4 weeks. Correspondingly, the surface volume ratio and the total porosity significantly increased in the I Cerament G, while the surface density was significantly decreased (Figure 4a).

The almost complete resolution of Cerament G was impressively visualized in the µ-CT postmortem. In comparison, the continuity of the bone substitute in the non-infected group was intact and the first remodeling processes were already visible (Figure 4b).

Cerament G density measurements (bone mineral density—BMD) showed a significant decrease in infected samples (Figure 4c). However, this was a further indication that the infection was responsible for the destruction of the bone substitute.

### 3.6. Cerament G Accelerates the Host Immune Response

**Figure 5:**
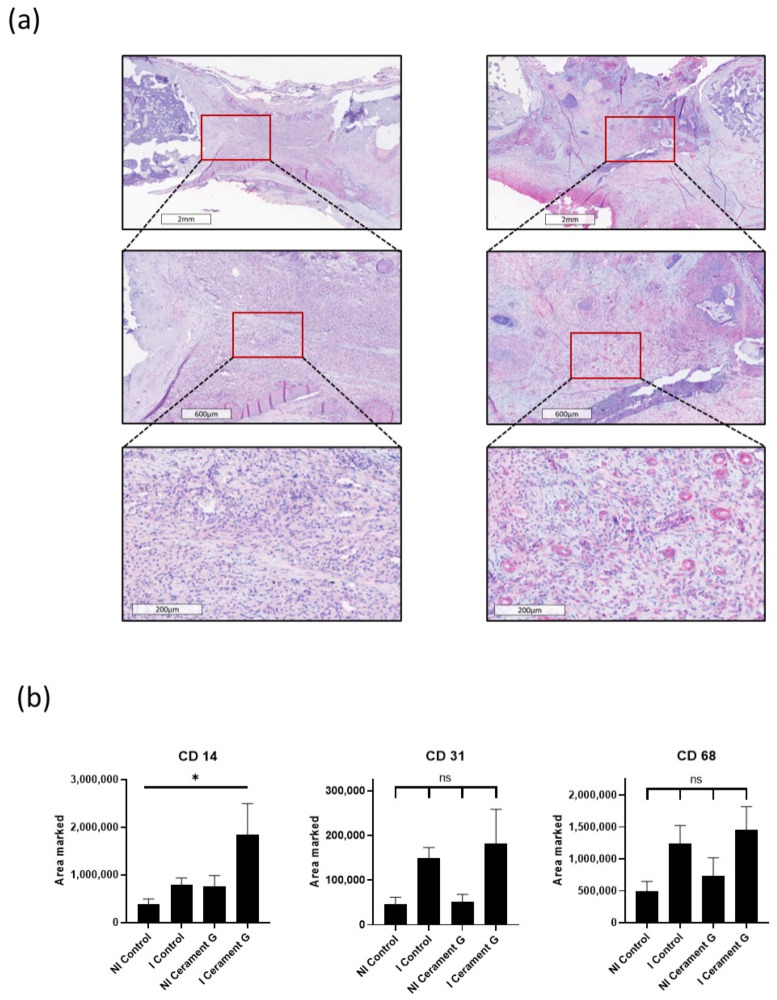
(**a**) CD14 staining, a marker for macrophages. Comparison of representative parts of the bone defect with magnification of the bone–defect border. (**b**) Significant increased infiltration of macrophages (CD14) in the I Cerament G compared with the NI Control, * = *p* < 0.05. CD31 staining, a marker for vascularization, and CD68 staining, a marker for macrophages, show no significant differences.

To compare the vascularization, immunohistochemical analysis using the Marker CD31 was performed. Hypervascularization was tendentially exhibited in the infected groups, with the strongest effect in the I Cerament group (Figure 5b).

To analyze the systemic inflammatory response, CD14 and CD68 stainings were prepared. Immunohistochemical staining of CD68 was the highest in the infected groups and thus analogous to the hypervascularization (Figure 5b). The CD14 marker was significantly increased in the I Cerament group compared with the NI Control group (Figure 5a). However, no differences could be detected between the I Control group and the NI Cerament group (Figure 5b).

### 3.7. The Infection Attenuates the Osteoinductive Effects of Cerament G

**Figure 6:**
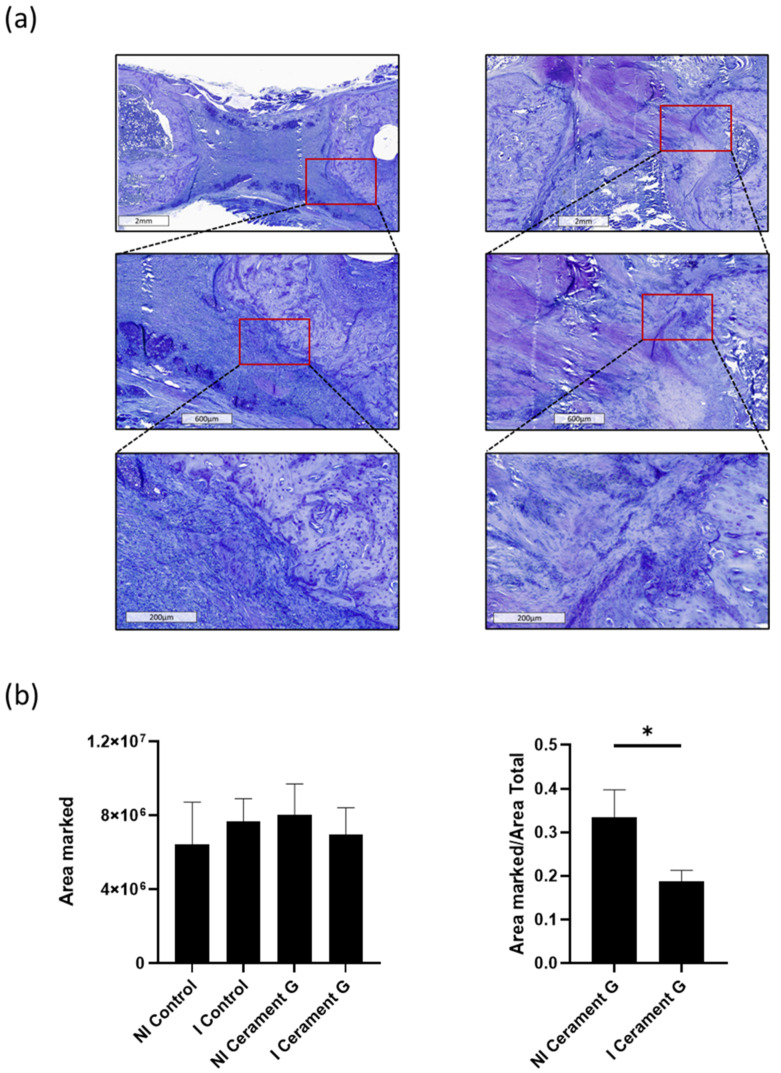
Toluidine staining shows significantly more invasion of toluidine positive osteoblasts in the defect of NI Cerament G compared to I Cerament G group. (**a**) + (**b**). * = *p* < 0.05.

Tolouidine staining of the bone substitute indicates a significant decrease in the NI Cerament group compared with the I Cerament group. This illustrates the osteoconductive potential of Cerament G and, at the same time, the compromise of this property in the infected situation (Figure 6).

## 4. Discussion

In a single center study from the UK, 96% of 100 patients with chronic osteomyelitis (78% Cierny and Mader Type III, 22% Cierny and Mader Type IV) were successfully treated using Cerament G in a single-stage procedure [19]. These promising results inspired other research groups to start similar studies. Niazi et al. successfully treated 73 patients with osteomyelitis in diabetic foot ulcers with Cerament G. The results were positive. With a mean follow-up of 10 months, bone healing was achieved in 90% of the patients [35]. Aljawadi et al. evaluated the outcome of 80 patients in the management of Open Gustilo-Anderson IIIB Fractures with single-stage “Fix and Flap” along with filling up the bone defect with Cerament G. Successful fracture union was achieved in 96.1% of patients, with a limb salvage rate of 96.25%. The infection rate was only 1.25%. However, only 5% of the patients had bone defects larger than 5 cm. In these cases, a limb shortening of 4 cm was performed, and 88.3% of the patients achieved primary fracture union [36]. Recently, contradictory results have also been published. In a retrospective analysis, 20 patients with chronic osteomyelitis and corticomedullary defects were treated with Cerament G or V, but 50% had to be revised due to wound healing disorders or persistent osteomyelitis [37].

These results demonstrate that reliable findings are difficult to achieve. Complicated by the heterogeneity of the patient population regarding acute vs. chronic infections, different bone defects, choice of surgical procedure, various microbial specimens, comorbidities, and, of course, the compliance of the patients. The presented study examined the role of HACaS+G (Cerament G) in a sequential non-union animal model [29] under non-infected and infected conditions. In this way, the osteoinductive, osteoconductive, and anti-infective efficacy of the antibiotic loaded bone substitute could be investigated in a controlled environment under infected and non-infected conditions.

The µ-CT analysis of the surrounding bone confirmed significant osteoinduction in the groups treated with Cerament G. This was reflected in the significantly increased bone volume and significantly decreased trabecular separation and porosity. It is important to note that this effect was observed independent of the presence or absence of an infection. The simultaneous increase in bone volume with increased density and decreased trabecular separation in the I Cerament G group compared with the I Control group indicates that osteoinduction is triggered by the bone substitute and not by the infection. This osteoinductive effect on the surrounding bone has also been demonstrated in previous animal [21,22] and clinical trials [23].

Clinical success in the treatment of non-unions is characterized by biomechanical stability [38]. No biomechanical stability and consequently bone consolidation could be demonstrated in any of the examined groups compared with the contralateral femora at the time of euthanasia. It is important to note that we did not expect a circular critical size defect to achieve biomechanical stability using the bone substitute in this short observation time. However, the bone substitute should be able to develop its full osteoconductive potential. The bone graft volume was significantly increased in the NI Cerament group compared with the I Cerament group at all time points. The osteoinductive effect in the non-infected setting can be illustrated by the significant increase in porosity and constant density over time in the µ-CT evaluation. This was also evident in the histological examination. In the overview staining, a noticeable migration of host cells into the bone substitute could be observed in NI Cerament G group. The migration of osteoblasts into the bone substitute was significantly increased in the NI Cerament G group compared with the I Cerament G group. In summary of the findings, Cerament G was not able to use its osteoinductive potential in the infected situation in our specific animal model setting. Similar findings were also made in other animal studies [39]. Regarding the significantly reduced density, the µ-CT analyses even allowed for the conclusion that Cerament G triggers the infection and thus its own degradation over time. This could be due to the decreasing antibiotic effect after 30 days [40].

From a critical point of view, the fact that antibiotics are not systematically administered, which is necessary in the treatment of infected non-unions and fracture-related infections [41], can be seen as a limitation of this study design. On the other hand, the antibacterial effect could not be attributed to the bone substitute. At the time of euthanasia, lower rates of secondary infections and bacterial contamination were observed in the NI Cerament group compared with the NI Control group. This suggests a prophylactic effect against infections of the bone substitute. At this point, the type of microbiological testing must be discussed. Microbiological swabs are inferior to other methods, such as tissue sampling, sonication, or polymerase chain reaction (PCR) [42,43,44]. However, with the combination of microbiological results and the evaluation of the µ-CT using the An and Friedmann score, a bone infection can be reliably diagnosed. The inoculated bacterial strain could be detected in all animals of the I Control group. In two animals of the I Cerament group, no germs could be detected 8 weeks after the second surgery. In one of these animals, the bacteria could not be detected at the time of the second surgery, which means inoculation must have failed. In the second animal, the An and Friedman score was >19, indicating that an infection had occurred, but could not be detected by microbiology. Consequently, eradication of the infection by Cerament G could not be achieved in any of the infected animals. This hypothesis is supported by the results of the histological examination. The migration of CD14-CD68-and CD31-positive cells into the bone substitute was tendentially more observed in the I Cerament group. This reflects the systematic immune response, which is also represented by hypervascularization. The results from µ-CT and histology are consistent and support the hypothesis that Cerament G tend to support the infection after delivery of gentamycin in this infected non-union model.

These and other previous studies [22] demonstrate the osteoinductive, osteoconductive, and at least anti-infective potential of Cerament G in the non-infected situation. The osteoconductive and anti-infective potential could not be demonstrated in the infected situation in this model. These findings are confirmed by the current clinical studies [37].

Further experimental and clinical studies are necessary in this field in order to use the bone substitute successfully. Further combinations of bone substitute with allogeneic or autologous bone grafting or with the additive use of osteoinductive pharmaceuticals such as bisphosphonates need to be explored. While the use of Cerament G appears to be promising for smaller cavitary bone defects even in the infected situations, other therapeutic strategies such as the use of the masquelet technique [45,46], bone transport by nail [47,48], or external fixator [49,50] are recommended for large circular defects.

## 5. Conclusions

HACaS+G (Cerament G) was used for the first time on a sequential infected non-union model on a rat femur. In the non-infected groups, the osteoinductive and osteoconductive efficiacy of Cerament G were significantly demonstrated. In the infected situation, only osteoinduction could be verified, whereas osteoconduction could not be observed. In contrast, degradation of the bone substitute occurred in the infected groups. Considering these aspects along with the lack of advantage in terms of bone consolidation and stability, our experimental data do not support the use of Cerament G for the specific treatment of infected non-unions with segmental bone defects.

## Figures and Tables

**Table 1:** The following groups were examined.

Groups	Procedure
1: Control group non-infected (NI Control) (*n* = 21) 2: Control group infected (I Control) (*n* = 14)	K-wire osteosynthesis with intramedullary application of 10 µL PBS (non-infected)/10^3^ KBE *S. aureus* in 10 µL PBS (infected) Debridement and re-osteosynthesis after 5 weeks with an angle-stable plate
3: Intervention group non infected (NI Cerament) (*n* = 16) 4: Intervention group infected (I Cerament) (*n* = 14)	K-wire osteosynthesis with intramedullary application of 10 µL PBS (non-infected)/10^3^ KBE *S. aureus* in 10 µL PBS (infected) Debridement and re-osteosynthesis after 5 weeks with an angle-stable plate and application of Cerament G to the bone defect.

## Data Availability

The datasets used and analyzed during the current study are available from the corresponding author upon reasonable request.
